# A DNA Barcode-Based RPA Assay (BAR-RPA) for Rapid Identification of the Dry Root of *Ficus hirta* (Wuzhimaotao)

**DOI:** 10.3390/molecules22122261

**Published:** 2017-12-18

**Authors:** Enwei Tian, Qianqian Liu, Haoting Ye, Fang Li, Zhi Chao

**Affiliations:** 1 School of Traditional Chinese Medicine, Southern Medical University, Guangzhou 510515, China; tianenwei@126.com (E.T.); yehaoting@163.com (H.Y.); lif0924@126.com (F.L.); 2 Affiliated Cancer Hospital & Institute of Guangzhou Medical University, Guangzhou 510095, China; liuqianqian@gzhmu.edu.cn

**Keywords:** DNA barcode-based recombinase polymerase amplification (BAR-RPA), rapid identification, Wuzhimaotao, Duanchangcao

## Abstract

**Background:** Wuzhimaotao (the dry root of *Ficus hirta*) is used as both medicine and food ingredient by the locals in areas around Nanling Mountains of China. Due to its very similar external morphologies with Duanchangcao (the root of *Gelsemium elegans*, which contains gelsemine that is extremely neurotoxic) and the associated growth of these two plants, incidents of food poisoning and even death frequently occur, resulting from the misuse of Duanchangcao as Wuzhimaotao. The aim of this study is to develop a fast, even, on-spot approach to identification of Wuzhimaotao. **Methods:** We used DNA barcode-based recombinase polymerase amplification (BAR-RPA) with species–specific primers targeting the internal transcribed spacer (ITS) region of the rDNA of *F. hirta.* BAR-RPA reaction time and temperature were optimized and the specificity and sensitivity of BAR-RPA species–specific primers were assessed. **Results:** This technique showed a high specificity and sensitivity to amplify the genomic DNA of *F. hirta* and allowed for rapid amplification (within 15 min) of the ITS region under a constant and mild temperature range of 37–42 °C without using thermocyclers. **Conclusions:** The BAR-RPA assay with a fast DNA extraction protocol provides a simple, energy-saving, and rapid method for identification of Wuzhimaotao in both laboratory and field settings.

## 1. Introduction

Wuzhimaotao is the dry root of *Ficus hitra* (*Moraceae* family, *Ficus* subgenus) [1], which is either a shrub or a small tree found in Southeast Asia (Indonesia), Northeast India and South China [2]. In traditional Chinese medicine (TCM), Wuzhimaotao as a herbal medicine has a sweet, pungent flavor; it is thought to produce such effects as strengthening the spleen, tonifying the lungs, removing dampness, and relaxing sinews and is used for treatment of spleen deficiency edema, weakness, coughing, tuberculosis, night sweats, and rheumatic arthralgia and for promoting postpartum milk production [3,4]. In addition to its medicinal values, Wuzhimaotao is also used as a popular food ingredient (especially in stewing soup) by the local residents in the areas around Nanling Mountains (i.e., Guangdong, Guangxi, Hunan, and Jiangxi Provinces) of China [5].

The most common adulterant of Wuzhimaotao is the toxic dry root of *Gelsemium elegans* (Loganiaceae) that contains the potent neurotoxin gelsemine. *Gelsemium elegans* is distributed in the same areas and often shares habitats with *F. hirta* [6]. As specimens of *F. hirta* and *G. elegans* often grow together and have very similar root morphologies, their differentiation can be difficult, and the mistaken use of Duanchangcao as the food ingredient Wuzhimaotao can very likely cause fatalities. To ensure food safety, it is necessary to develop a rapid and accurate method for differentiation between Wuzhimaotao and its adulterant Duanchangcao (the images of both living plants and processed dry roots are shown in Figure S1).

DNA molecular marker technologies have long been used to identify species based on the different characteristics of the DNA fragments [7,8]. These highly sensitive technologies produce reliable results that are less affected by environmental factors, sample forms, or the experience of operators and thus have great potentials in the identification of TCM herbal drugs [9]. The technologies of DNA molecular identification of medicinal herbs in TCM has evolved through three stages: from restriction fragment length polymorphisms (RFLPs), to random amplification of polymorphic DNA (RAPD), and to DNA barcoding [10]. DNA barcoding based on sequence analysis has been used for the identification of TCM herbal drugs [11,12]. However, these methods are still insufficient for herbal drug authentication due to their complexity and inaccuracy [13], as they rely heavily on PCR amplification, which is a time-consuming (usually at least 2–3 h) process and requires the use of sophisticated equipment (thermocycler) and complex procedures. These limitations confine the use of the current DNA molecular approaches to TCM drug identification to specialized laboratories.

In the past decade, isothermal amplification techniques have evolved rapidly, including loop-mediated isothermal amplification (LAMP) and recombinase polymerase amplification (RPA) techniques [14,15,16]. These isothermal amplification techniques do not require a thermocycler and yield readily readable results [17,18]. These techniques have been applied in the diagnosis of bacterial, viral, or parasite infections and in food and cosmetics safety control [19]. In 2008, Yohei et al. (2008) successfully designed a LAMP primer for 18S rRNA gene for differentiation of Gingseng Radix et Rhizoma from its adulterants Panacis Japonici Rhizoma and Glycyrrhizae Radix [20]. Jiang et al. (2011) similarly reported success in identifying Herba Taraxaci from its adulterants using the LAMP technique [21]. LAMP allows fast amplification without using thermocyclers, but it requires four specific primers for six areas of the targeted gene, and the amplification time is comparatively long (about 60 min).

The DNA barcode-based RPA (BAR-RPA) technique uses recombinase, polymerase, and single-stranded binding protein (SBB) to replace the unwinding chain process of the traditional PCR technique. It achieves rapid amplification (around 15 min) of the genetic marker under a constant temperature of 37–39 °C without using thermocyclers [22]. When coupled with a rapid DNA extraction method, the BAR-PRA technique can provide an efficient detection method for use in different scenarios including food and drug administration, forensic medicine, and laboratory examinations; the technique has been used in molecular assays for digital, microfluidics, agriculture, veterinary, and defense applications [23,24,25,26]. However, no studies have yet documented the use of the BAR-RPA technique in the identification of TCM drugs. In this study, we established a BAR-RPA assay and evaluated its performance for the identification of Wuzhimaotao and its adulterant Duanchangcao. 

## 2. Results

### 2.1. Optimal Reaction Temperature and Time for BAR-RPA 

**Figure 1:**
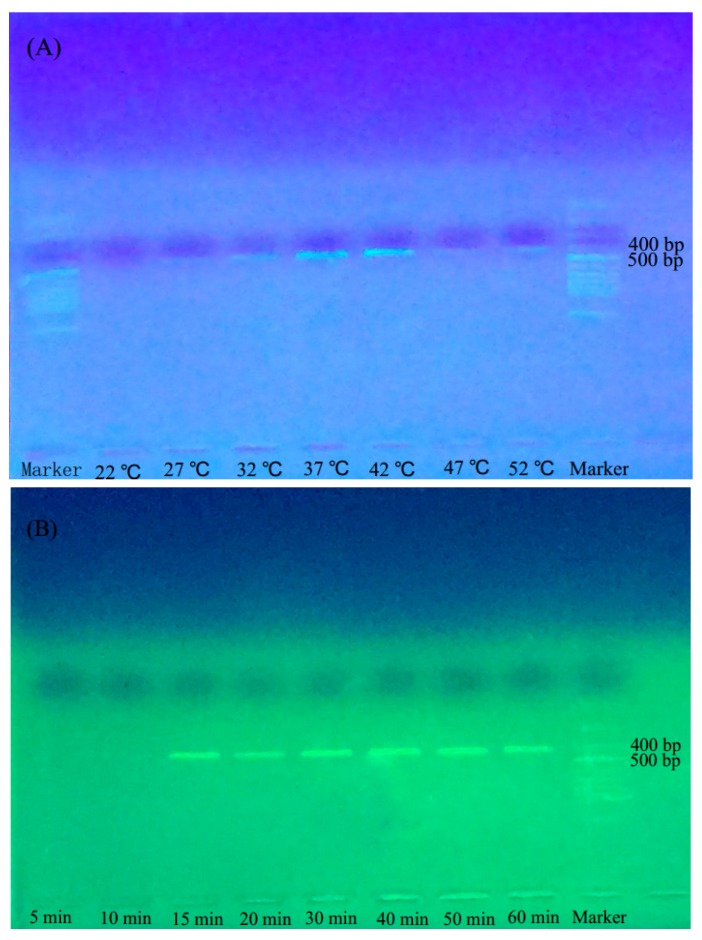
Results of optimal temperature (**A**) and time (**B**) for the DNA barcode-based recombinase polymerase amplification (BAR-RPA) reaction. (**A**) The optimization of temperature for the BAR-RPA (range: 22–52 °C); (**B**) The optimization of time for the BAR-RPA (range: 5–60 min). Lane Marker: 50 bp DNA ladder.

The results of the amplification showed that, within the temperature range of 27–52 °C, the *F. hirta* sample WZS-2 could be successfully amplified, and the optimal temperature range for the PRA was 37–42 °C (Figure 1A). We chose the temperature of 38 °C as the optimal temperature to optimize the reaction time of BAR-RPA. The results of gel electrophoresis showed that the amplification signals appeared at 15 min after the start of the reaction. Amplifications for 5–60 min all yielded single and stable bands, and the shortest BAR-RPA reaction time was 15 min (Figure 1B).

### 2.2. Specificity and Sensitivity Assessment of BAR-PRA

**Figure 2:**
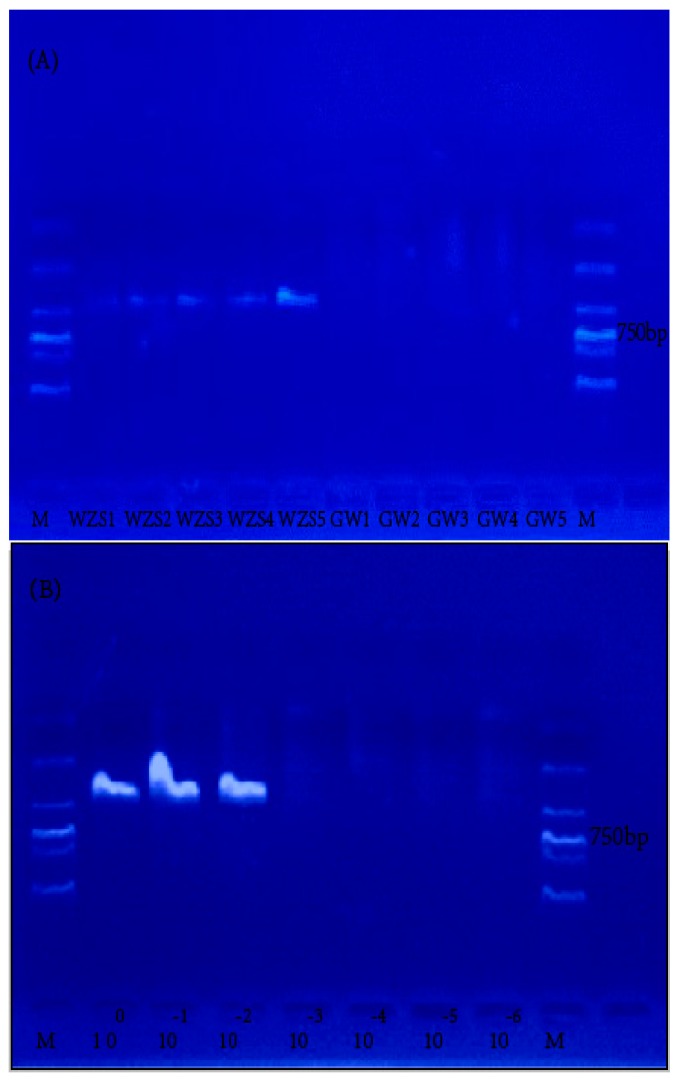
Results of specificity and sensitivity for the BAR-RPA assays. (**A**) Five samples for each species, *F. hirta* (WZS-1~WZS-5) and *G. elegans* (GW-1~GW-5) were tested for the specificity of the BAR-RPA assays; (**B**) Sensitivity of the BAR-RPA was assessed with the WZS-2 sample (dilutions: 10^0^, 10^−1^, 10^−2^, 10^−3^, 10^−4^, 10^−5^, and 10^−6^ ng/µL).

We tested the specificity of the designed species–specific primers using 10 samples (5 for each species) (Table 1), and additional 12 samples (6 for each species) were also tested (see Table S1 and Figure S2). The DNA concentration of the 10 extracts ranged from 30 to 50 ng/µL, and the absorbance (*A*) ratio at 260 and 280 nm (*A*_260_/*A*_280_) was 1.78–1.95. The ITS sequence was successfully amplified from all five *F. hirta* samples with a clearly visible band on the gel. Amplification of the *G. elegans* samples all yielded negative results, suggesting a high specificity of the primers to *F. hirta* (Figure 2A). 

In assessing the assay sensitivity, we found that the BAR-PRA assay was capable of amplifying the ITS sequence when the genomic DNA of the WZS-2 sample was diluted from 36.8 to 36.8 × 10^−2^ ng/µL, but failed when double-diluted of 36.8 × 10^−2^ ng/µL (18.4 ng/µL). This result suggested that the lowest detection limit of this BAR-RPA assay was 36.8 × 10^−2^ ng/µL (0.368 ng/µL) (Figure 2B).

### 2.3. Authenticity Identification of *Wuzhimaotao* in the Market

**Figure 3:**
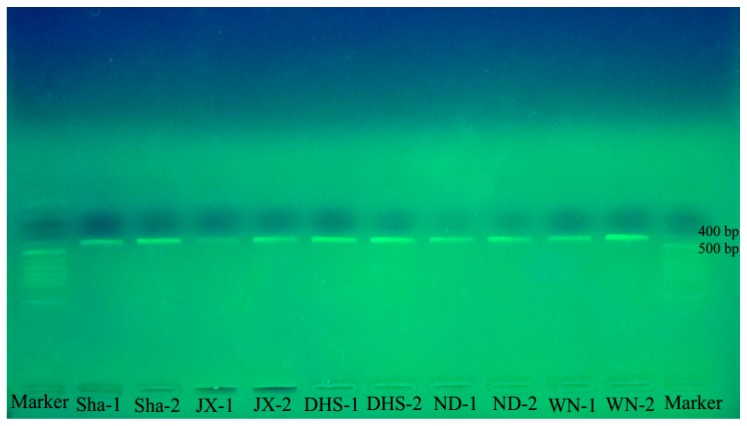
The BAR-RPA assays for identification of Wuzhimaotao in market samples. Lane Marker: 50 bp DNA Ladder. Lanes 2–11: 10 crude drug samples of Wuzhimaotao from 5 medicinal material markets (Sha, JX, DHS, ND, and WN).

The genomic DNA concentration extracted from the 10 crude drug samples of Wuzhimaotao ranged from 20 to 50 ng/µL (*A*_260_/*A*_280_: 1.71–1.97), which met the requirements for the subsequent BAR-PRA assay. The amplification results showed that a band of 412 bases was successfully amplified from all the 10 samples (Table 2; Figure 3), demonstrating the validity of the specific primers in rapid identification of Wuzhimaotao.

## 3. Discussion

In this study, we established a BAR-RPA assay coupled with rapid DNA extraction for the identification of Wuzhimaotao and its adulterant Duanchangcao. In conventional molecular approaches, DNA extraction is time-consuming and involves complex sample treatments. The Tris-neutralization method allows effective and fast DNA extraction from such plants as corn, wheat, rice, and peanut [27,28], and we modified the Tris-neutralization method by treating the samples with 0.5 mol/L NaOH to cause cell lysis and the full release of DNA from the cells [29]. DNA extraction using this method resulted in a DNA concentration of >20 ng/µL with an *A*_260_/*A*_280_ range of 1.7–1.97, which well met the needs of the PRA assay. Compared with other methods [30], this modified approach is simple and fast (within 5 min) without using toxic reagents, and it is particularly suitable for on-site applications.

To choose a target barcode, we conducted preliminary experiments with common barcode markers, notably ITS, psbA-trnH, rbcL, and matK. These are widely recognized for their usefulness in species identification [11]. ITS is characterized by a high rate of evolution and features many variable and informative sites [31]. We chose the ITS genetic marker for amplification in our BAR-RPA assay. For the design of the primers, measures were taken to ensure that the primers had a GC content of 40–60% with a sequence length of 28–34 bases without a secondary structure or repeat sequences [32,33,34]. The amplification results of the DNA extracts from *F. hirta* and *G. elegans* samples demonstrated the satisfactory specificity of the designed primers (Figure 2A).

The temperature and time of amplification are the two most important parameters in the BAR-RPA assays. Unlike other amplification techniques, BAR-RPA can reduce the amplification time to 15–25 min under relatively stable and mild temperatures of 37–39 °C [33,34,35]. In conventional PCR, a thermocycler is required for controlling the temperature of the reaction, and the procedure can take from 2–3 h up to a full day. Even the isothermal amplification technique of LAMP requires high reaction temperatures ranging from 60 to 65 °C, and the reaction can last for 60 min [14]. The BAR-RPA assay is much easier and more rapid than conventional PCR and LAMP. Our results showed that the optimal amplification temperature range was 37–42 °C for BAR-RPA detection as described in the detection of *Aspergillus fumigatus* [36]; surprisingly, the samples could be amplified even at room temperature (27 °C; Figure 1A). Therefore, the BAR-RPA assay requires only a heating module when the environmental temperature is below 25 °C. We found that the amplification time in the RPA assay can be reduced to 15 min, as extending the amplification time to 20 or 60 min did not produce any significant difference in the results; we therefore chose 15 min as the optimal reaction time in the BAR-RPA assay (Figure 1B). By combining BAR-RPA with the rapid DNA extraction method, the entire BAR-RPA assay for the detection of Wuzhimaotao can be completed within 20 min and can even be performed in an outdoor setting.

In the BAR-RPA assay, results of the amplification reaction can be assessed using gel electrophoresis, fluorescent probes, or lateral-flow strips [33,37]. We used agarose gel electrophoresis to minimize the cost. Compared with fluorescent probes and lateral-flow strips, gel electrophoresis is more suitable in the BAR-RPA assays for rapid identification of TCM in not so well-equipped laboratories. The total cost to process a sample via such methods as DNA extraction, amplification, and detection is approximately $5 USD.

The result of our sensitivity assessment showed that the BAR-RPA assay we established had a detection limit of 10^−2^ ng/µL of genomic DNA samples, which is consistent with those of previously reported RPA assays for identification of *Penaeus stylirostris* and *Francisella tularensis* [25,36,38]. In testing the commercially available samples of Wuzhimaotao crude drugs, the RPA assay yielded positive results for all 10 samples, and the 10 RPA products were also sequenced and Blasted in the Barcode of Life Data systems and Genbank (similarity comparison with *F. hirta*: >99%; sequence coverage: 100%) [39]. All of this suggests that this BAR-RPA assay is highly specific in authenticity identification of Wuzhimaotao with the designed primers. Although the investigated samples are all genuine Wuzhimaotao (Table 2), we have to consider the sampling bias. In the Chinese herbal medicine market, the Food and Drug Administration should enact strict supervision (by having regular spot checks and increasing the number of samples that are checked) of the quality of Wuzhimaotao, which would avoid mistaking Duanchangcao for Wuzhimaotao for stewing soup or medicine.

## 4. Materials and Methods

### 4.1. Materials

Root samples of 11 specimens of *Ficus hirta*, a species that is used as Wuzhimaotao in Chinese markets, were collected from the Wuzhi Mountain in Conghua district of Guangzhou, Guangdong province of China. Eleven root samples of *Gelsemium elegans* were also collected in the same area (Table 1 and Table S1). All samples were identified by Prof. Zhi Chao from School of Traditional Chinese Medicine, Southern Medical University. All voucher specimens were deposited in the herbarium of Southern Medical University (Guangzhou, China). Ten commercial samples of Wuzhimaotao crude drugs were purchased from 5 medicinal material markets in China to test the identification accuracy of the BAR-RPA assay.

### 4.2. Methods

#### 4.2.1. DNA Extraction

The total genomic DNA was extracted from a 30 mg sample of dry roots using a modified Tris-neutralization method [29], in which an extraction buffer containing 0.5 mol/L NaOH, 1% polyvinyl polypyrrolidone (PVP) and 1% Triton × 100 was used. Extraction of the genomic DNA was performed through the following procedures: The extraction buffer (20 μL) was added in a 1.5 mL EP tube containing the sample powder. This tube was vortexed for 10–15 s followed by boiling for 10–15 s; 80 μL Tris-HCl (0.1 mol/L, pH 8.0) was then added into the mixture, vortexed gently and centrifuged for 2 min at 12,000 r/min; the supernatant was collected for later use. The quality and quantity of the exacted DNA samples were assessed using a NanoDrop 1000 UV/Vis spectrophotometer (Thermo Scientific, Wilmington, DE, USA) and 1.2% agarose gel electrophoresis.

#### 4.2.2. Primer Design

The key to a successful BAR-RPA run is the design of suitable species–specific primers. We chose the internal transcribed spacer (ITS) region with abundant variation sites to design the species–specific primers. Respectively, the 5 and 3 full-length ITS sequences of *F. hirta* (GenBank accession No. JQ773900, JQ773899, JQ773898, JQ773897, and AY730127) and *G. elegans* (HG004870, KF022348 and KF022347) were downloaded from GenBank (http://www.ncbi.nlm.nih.gov/) and then, to identify the specific variation sites, aligned with ClustalX and analyzed with MGEA 5.1 software. Quality control for the sequences downloaded from GenBank and our new sequence was conducted in accordance with five simple quality control guidelines for establishing basic authenticity and reliability [40]. The specific primers for BAR-RPA were designed using Primer3 (v.0.4.0, Whitehead Institute for Biomedical Research, Cambridge, MA, USA) according to the descriptions in TwistDx manual [32,41]. Based on the ITS sequence of *F. hirta* (GenBank accession No. JQ773900), two pairs of primers were designed, and one of them was selected:

RPA-ITS-F: 5′-TCAAGGAAAGACAACGAGACGATCCCAGCC-3′;
RPA-ITS-R: 5′-CGACTACCTGTTGCCAAGACGACGTGACAG-3′.

This pair of primers was expected to produce an amplicon of 412 bases targeting a region (position: 131 to 542, including partial ITS1, 5.8 s and partial ITS2) of the ITS region. Primer sites of this pair of species–specific primers are shown in Table S2.

#### 4.2.3. Amplification and Purification

A TwistAmp Basic kit (TwistDX, Cambridge, UK) was used for BAR-RPA. BAR-RPA reactions were established by reconstituting the supplied freeze-dried reaction pellets with a rehydration solution consisting of 1× TwistAmp Basic rehydration buffer (provided with the kit), 10 μM amplification primers, and the template (distilled water was added to make a total volume of 47.5 μL per sample). The reaction was initiated by the addition of 2.5 μL of magnesium acetate solution (provided with the kit), and the final reaction volume was 50 μL per sample [29]. The amplification product was purified using the Universal Purification Kit (Tiangen, Beijing). The BAR-RPA purified products were detected by 2% agarose gel electrophoresis and visualized under ultraviolet light.

#### 4.2.4. Optimization of BAR-RPA Reaction Temperature and Time 

To optimize the temperature and duration for the BAR-RPA reaction, we tested different temperature settings for amplifying the genomic DNA from 2 μL of a *F. hirta* sample (WZS-2). BAR-RPA reactions with the specific primers were carried out in a portable incubator or a thermostat at a temperature gradient of 22, 27, 32, 37, 42, 47, and 52 °C for 25 min. The optimal temperature (or range) was determined by observing the results of the gel electrophoresis. We subsequently optimized the amplification time with a gradient of 5, 10, 15, 20, 30, 40, 50, and 60 min at the optimal temperature using the same sample.

#### 4.2.5. Specificity and Sensitivity Assessment of the BAR-RPA Assay

The specificity of the assay was evaluated using genomic DNA templates from *F. hirta* samples (WZS-1, WZS-2, WZS-3, WZS-4, and WZS-5) and *G. elegans* samples (GW-1, GW-2, GW-3, GW-4, and GW-5). In each BAR-RPA reaction, 2 µL of the genomic DNA was used as the template for amplification with the optimal time and temperature setting. Observations of amplification signals in *F. hirta* samples but not in *G. elegans* were considered to determine the specificity of the primers.

For assessing the sensitivity of the BAR-RPA assay, we prepared serial dilutions (10^0^, 10^−1^, 10^−2^, 10^−3^, 10^−4^, and 10^−5^ ng/µL) of the genomic DNA of the *F. hirta* sample WZS-2 with double-distilled H_2_O. In each reaction, 2 µL of the diluted DNA sample was used as the template to test the lowest detection limit of BAR-RPA.

#### 4.2.6. Identification of Wuzhimaotao Crude Drugs Using BAR-PRA

The accuracy of BAR-RPA with the species–specific primers for identification of Wuzhimaotao was assessed using 10 crude drugs samples bought from 5 medicinal material markets in different provinces in China (2 from each market; Table 2) between April and August in 2015. BAR-RPA was performed with the optimized temperature and time setting.

## 5. Conclusions

In this study, we developed a BAR-RPA assay that showed good sensitivity and specificity for authenticity identification of Wuzhimaotao. Coupled with the rapid DNA extraction method, this rapid BAR-RPA assay is particularly suitable for TCM drug identification in field settings and can be used for quality control of TCM drugs.

## Figures and Tables

**Table 1:** Sample information and results obtained for the specificity of the BAR-RPA primers.

Name of Medicinal Herb	Sources	Code	Location	Results
Wuzhimaotao	*Ficus hirta*	WZS-1	Conghua, Guangdong	P
Wuzhimaotao	*Ficus hirta*	WZS-2	Conghua, Guangdong	P
Wuzhimaotao	*Ficus hirta*	WZS-3	Conghua, Guangdong	P
Wuzhimaotao	*Ficus hirta*	WZS-4	Conghua, Guangdong	P
Wuzhimaotao	*Ficus hirta*	WZS-5	Conghua, Guangdong	P
Duanchangcao	*Gelsemium elegans*	GW-1	Conghua, Guangdong	N
Duanchangcao	*Gelsemium elegans*	GW-2	Conghua, Guangdong	N
Duanchangcao	*Gelsemium elegans*	GW-3	Wanning, Hainan	N
Duanchangcao	*Gelsemium elegans*	GW-4	Conghua, Guangdong	N
Duanchangcao	*Gelsemium elegans*	GW-5	Conghua, Guangdong	N

P: Successful amplification with BAR-RPA specific primers. N: Failure of amplification.

**Table 2:** Authenticity identification of the crude drug of Wuzhimaotao from different sources.

Code	Places of Production	Results of Identification
Sha-1	Shaxian, Fujian	+
Sha-2	Shaxian, Fujian	+
JX-1	Jinggangshan, Jiangxi	+
JX-2	Jinggangshan, Jiangxi	+
DHS-1	Dinghushan, Guangdong	+
DHS-2	Dinghushan, Guangdong	+
ND-1	Ningde, Fujian	+
ND-2	Ningde, Fujian	+
WN-1	Wanning, Hainan	+
WN-2	Wanning, Hainan	+

“+” indicated the crude drug is genuine Wuzhimaotao.

## Supplementary Materials

Supplementary materials are available on line. Figure S1: The living plants (*Ficus hirta*, A; *Gelsemium elegans*, C) and the form of processed medicinal samples (Wuzhimaotao, B; Duangchangcao, D), Figure S2: Results of specificity and sensitivity analysis for the BAR-RPA assays, Table S1: The 12 samples (6 for each species) information and tested results of the specificity of the BAR-RPA primers, Table S2: Primer sites of species-specific primers RPA-ITS-F and RPA-ITS-R designed for the identification of *Ficus hirta* based on ITS sequences among the two species.
