# Supplementary material for: A DNA Barcode-Based RPA Assay (BAR-RPA) for Rapid Identification of the Dry Root of Ficus hirta (Wuzhimaotao)
Source: Molecules. 2017 Dec 18;22(12):2261. doi: 10.3390/molecules22122261 (PMC6149672; doi:10.3390/molecules22122261)
Supplement: Supplementary file 1 [file molecules-22-02261-s001.pdf]

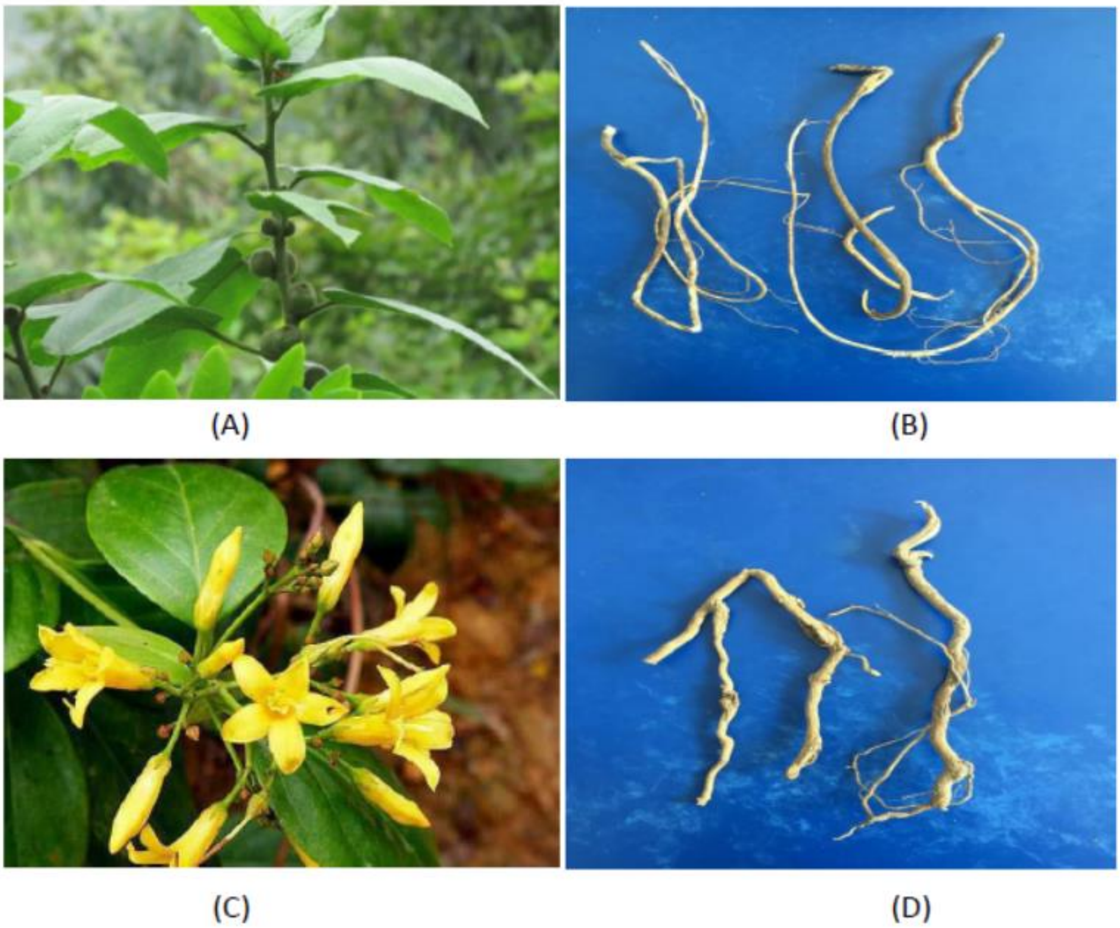

Figure S1. The living plants (*Ficus hirta*, A; *Gelsemium elegans*, *C*) and the form of processed medicinal samples (*Wuzhimaotao*, B; *Duangchangcao*, D)

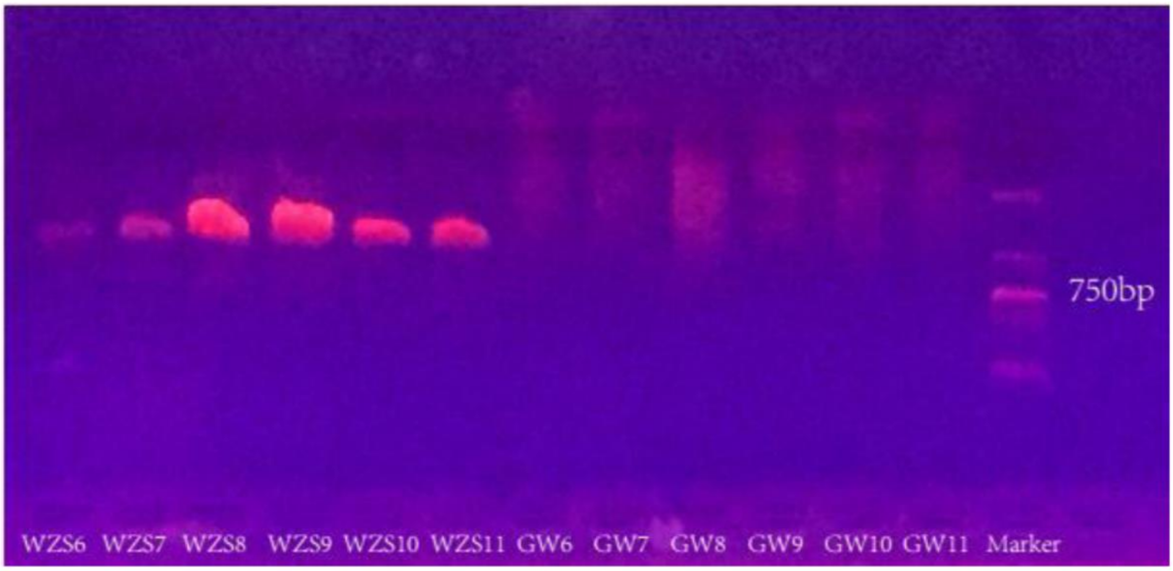

Figure S2. Results of specificity and sensitivity analysis for the BAR-RPA assays. The 6 samples for each species, *F. hirta* (WZS6~WZS11) and *G. elegans* (GW6~GW1) were tested for the specificity of the BAR-RPA assays.

Table S1. The 12 samples(6 for each species) information and tested results of the specificity of the BAR-RPA primers

Name of medicinal herb	Sources	Code	Location	Results
Wuzhimaotao	*Ficus hirta*	WZS-6	Conghua,Guangdong	P
Wuzhimaotao	*Ficus hirta*	WZS-7	Conghua,Guangdong	P
Wuzhimaotao	*Ficus hirta*	WZS-8	Conghua,Guangdong	P
Wuzhimaotao	*Ficus hirta*	WZS-9	Conghua,Guangdong	P
Wuzhimaotao	*Ficus hirta*	WZS-10	Conghua,Guangdong	P
Wuzhimaotao	*Ficus hirta*	WZS-1	Conghua,Guangdong	P
Duanchangcao	*Gelsemium elegans*	GW-6	Conghua,Guangdong	N
Duanchangcao	*Gelsemium elegans*	GW-7	Conghua,Guangdong	N
Duanchangcao	*Gelsemium elegans*	GW-8	Conghua,Guangdong	N
Duanchangcao	*Gelsemium elegans*	GW-9	Conghua,Guangdong	N
Duanchangcao	*Gelsemium elegans*	GW-10	Conghua,Guangdong	N
Duanchangcao	*Gelsemium elegans*	GW-11	Conghua,Guangdong	N

Note: "P" indicates successful amplification with BAR-RPA specific primers; "N", failure of amplification.

Table S2. Primer sites of species-specific primers RPA-ITS-F and RPA-ITS-R designed for the identification of *Ficus hirta* based on ITS sequences among the two species

	131																												
Ficus hirta-JQ773900	T	C	A	A	G	G	A	A	A	G	A	C	A	A	C	G	A	G	A	C	G	A	T	C	C	C	A	G	C
Ficus hirta-JQ773899	•	•	•	•	•	•	•	•	•	•	•	•	•	•	•	•	•	•	•	•	•	•	•	•	•	•	•	•	•
Ficus hirta-JQ773898	•	•	•	•	•	•	•	•	•	•	•	•	•	•	•	•	•	•	•	•	•	•	•	•	•	•	•	•	•
Ficus hirta-JQ773897	•	•	•	•	•	•	•	•	•	•	•	•	•	•	•	•	•	•	•	•	•	•	•	•	•	•	•	•	•
Ficus hirta-AY730127	•	•	•	•	•	•	•	G	•	•	•	•	C	•	•	•	•	•	•	•	•	•	•	•	•	•	•	•	•
Gelsemium elegans-HG004870	C	•	•	•	•	•	•	•	•	A	C	•	G	•	A	A	C	•	G	A	T	T	G	•	•	A	T	C	•
Gelsemium elegans-KF022348	C	•	•	•	•	•	•	•	•	A	C	T	•	•	A	A	C	•	G	A	T	T	G	•	•	A	T	C	•
Gelsemium elegans-KF022347	C	•	•	•	•	•	•	•	•	A	C	T	•	•	A	A	C	•	G	A	T	T	G	•	•	A	T	C	•
	513																												
Ficus hirta-JQ773900	C	T	G	T	C	A	C	G	T	C	G	T	C	T	T	G	G	C	A	A	C	A	G	G	T	A	G	T	C
Ficus hirta-JQ773899	•	•	•	•	•	•	•	•	•	•	•	•	•	•	•	•	•	•	•	•	•	•	•	•	•	•	•	•	•
Ficus hirta-JQ773898	•	•	•	•	•	•	•	•	•	•	•	•	•	•	•	•	•	•	•	•	•	•	•	•	•	•	•	•	•
Ficus hirta-JQ773897	•	•	•	•	•	•	•	•	•	•	•	•	•	•	•	•	•	•	•	•	•	•	•	•	•	•	•	•	•
Ficus hirta-AY730127	•	•	•	•	•	•	•	•	•	•	•	•	•	•	•	•	•	•	•	•	•	•	•	•	•	•	•	•	•
Gelsemium elegans-HG004870	•	—	•	C	G	•	•	•	G	•	•	•	•	A	C	•	A	•	•	•	G	T	•	•	•	G	•	•	T
Gelsemium elegans-KF022348	•	C	•	C	G	•	•	•	G	•	•	•	•	A	C	•	A	•	•	•	G	T	•	•	•	G	•	•	T

Gelsemium elegans-KF022347	•	C	•	C	G	•	•	•	G	•	•	•	•	A	C	•	A	•	•	•	G	T	•	•	•	G	•	•	T

Note: The forward primer (RPA-ITS-F): 5'-TCAAGGAAAGACAACGAGACGATCCCAGCC-3', positioning from 131 to 160; the reverse primer(RPA-ITS-R): 5' -CGACTACCTGTTGCCAAGACGA CGTGACAG- 3', positioning from 513 to 542.
